# Supplementary material for: Comparative Transcriptome Analysis Reveals Sex-Based Differences during the Development of the Adult Parasitic Wasp Cotesia vestalis (Hymenoptera: Braconidae)
Source: Genes (Basel). 2021 Jun 10;12(6):896. doi: 10.3390/genes12060896 (PMC8228208; doi:10.3390/genes12060896)
Supplement: Supplementary file 1 [file genes-12-00896-s001.zip › genes-1188101-supplementary-1.pdf]

Table S2 RNA-Seq sequencing data summary.

Sample	Raw Reads No.	Clean Reads No.	Q30 (%)	Mapped Reads No.	Mapping Rate (%)	Assembled transcripts No.
PM2d_1	44,033,388	43,759,232	92.22	41,590,220	95.04	22,300
PM2d_2	46,697,504	46,500,602	92.74	44,379,153	95.44	22,608
PM2d_3	47,142,052	46,852,494	93.62	44,757,492	95.53	21,691
PF2d_1	41,933,846	41,139,782	92.78	38,991,656	94.78	21,005
PF2d_2	45,680,646	44,914,330	92.81	42,500,418	94.63	21,691
PF2d_3	42,303,128	41,544,724	91.93	39,057,644	94.01	21,635
PM3d_1	46,637,386	46,443,740	93.85	44,586,508	96.00	19,809
PM3d_2	45,498,354	45,291,078	93.82	43,455,865	95.95	19,969
PM3d_3	46,755,536	46,546,310	92.76	44,554,400	95.72	20,263
PF3d_1	44,276,248	43,984,210	93.28	42,130,921	95.79	20,334
PF3d_2	46,723,798	46,492,992	92.96	44,518,135	95.75	20,577
PF3d_3	46,372,024	46,061,682	92.52	44,056,046	95.65	20,267
PM4d_1	43,671,708	43,385,016	92.28	41,231,569	95.04	19,850
PM4d_2	44,121,254	43,873,038	93.11	41,955,730	95.63	19,680
PM4d_3	44,794,120	44,503,622	93.65	42,490,117	95.48	20,021
PF4d_1	44,237,218	44,045,830	92.93	42,140,802	95.67	21,185
PF4d_2	46,982,982	46,785,592	93.27	44,695,703	95.53	21,829
PF4d_3	44,201,712	43,916,196	93.44	41,982,303	95.60	21,312
AM1d_1	46,543,036	46,230,626	93.02	44,111,210	95.42	22,079
AM1d_2	44,875,604	44,653,156	93.42	42,604,337	95.41	21,421
AM1d_3	44,201,930	43,922,388	92.42	41,866,117	95.32	21,737
AF1d_1	44,327,068	44,094,826	93.59	42,148,204	95.59	23,257
AF1d_2	46,001,024	45,740,928	93.36	43,667,483	95.47	23,452
AF1d_3	44,816,536	44,620,214	93.27	42,628,625	95.54	23,261

Table S3 Data overview of RNA-Seq and Iso-Seq mapped to *C. vestalis* genome.

Sequencing pattern	Transcripts No.	Non-redundant transcripts No.[1]	Gene coverage	Novel non-redundant transcripts No.
RNA-Seq	32,102	22,196	13,388 (99.98%)	14,466
Iso-Seq	77,590	76,773	7,629 (56.97%)	11,849

[1]Non-redundant transcripts No.: Remove the number of duplicate isoforms by CD-hit-est (default parameters).

Table S4 PacBio single-molecular long-read sequencing data summary.

Category	1-2kb	2-3kb	3-6kb	Total
Circular-consensus sequences(CCSs)	157,628	175,506	79,090	412,224
Number of poly-A reads	114,990	110,511	51,671	277,172
Number of filtered short reads	8,445	7,994	1,473	17,912
Non-full-length(NFL)	54,784	88,194	40,953	183,931
Full-length	94,399	79,318	36,664	210,381
Full-length non-chimeric(FLNC)	92,921	78,624	36,410	207,955
Average FLNC length(bp)	1,471	2,566	3,482	7,519
Number of consensus isoforms	33,112	25,553	19,473	78,138
Average consensus isoforms read length	1,489	2,648	3,626	7,763
Number of polished high-quality isoforms	16,964	10,461	6,839	34,264
Number of polished low-quality isoforms	16,148	15,090	12,634	43,872

Table S12 Alternative splicing genes identified in RNA-Seq data of different developmental stages and sexes in *C. vestalis.*

Type	PM2d	PM3d	PM4d	AM1d	PF2d	PF3d	PF4d	AF1d	Total
A3	1,385	1,265	1,267	1,334	1,349	1,280	1,347	1,507	1,835
A5	1,410	1,315	1,299	1,393	1,398	1,346	1,397	1,505	1,857
AF	348	317	310	321	314	313	338	363	574
AL	94	86	83	88	97	84	90	98	141
RI	397	354	327	356	375	345	352	388	692
SE	506	483	490	515	526	474	517	614	770
MX	32	33	32	31	34	33	33	36	47
Total	3,198	2,960	2,927	3,088	3,120	2,964	3,102	3,402	4,192

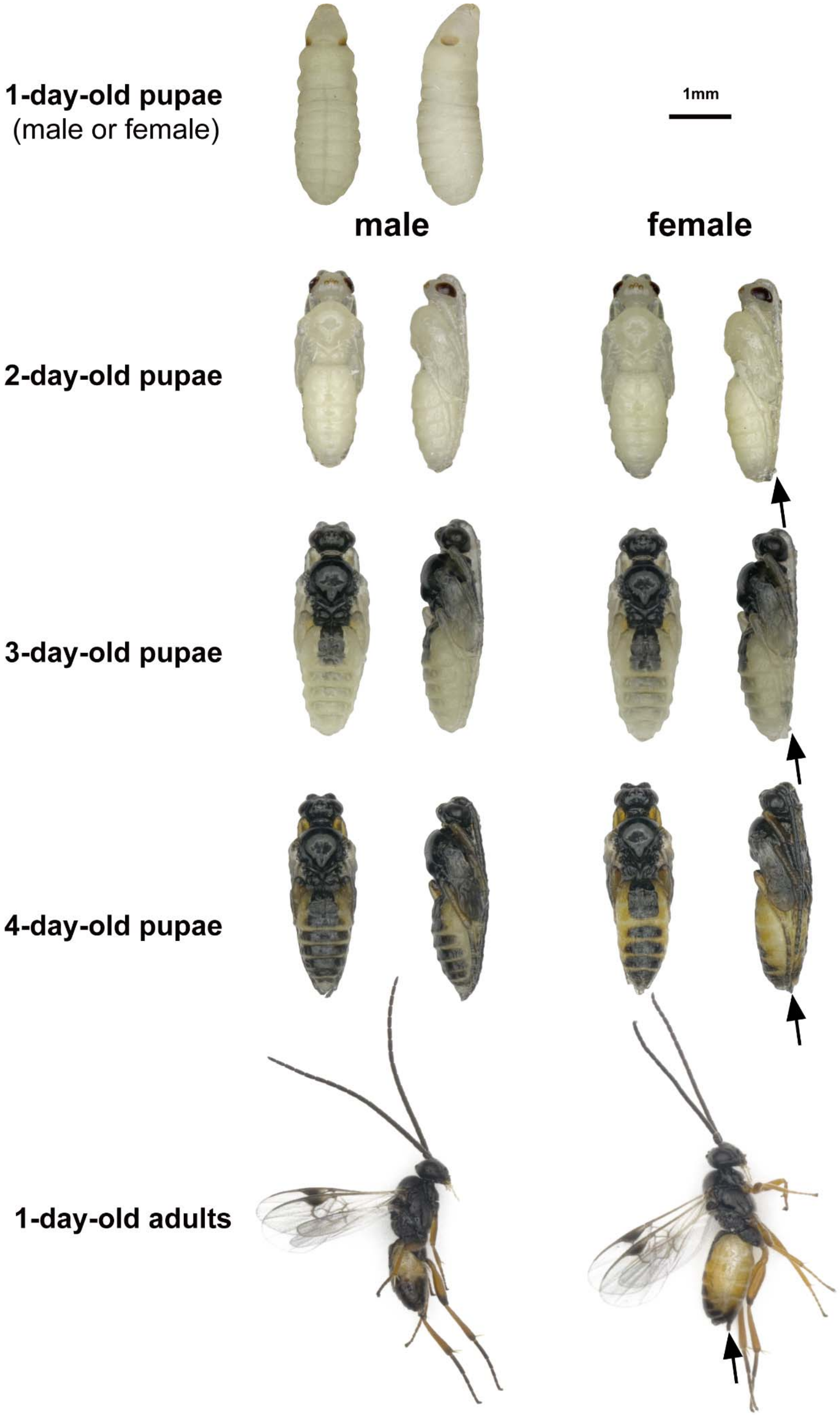
1-day-old pupae
(male or female)
1mm
male
female
2-day-old pupae
3-day-old pupae
4-day-old pupae
1-day-old adults

Figure S1. Representative images of *C. vestalis* different developmental stages of pupae and adults both sexes (male: left, female: right). The pupal stage is shown in front and side views. The black arrow indicates the valvula of ovipositor and mature ovipositor, respectively. Scale bar: 1 mm.

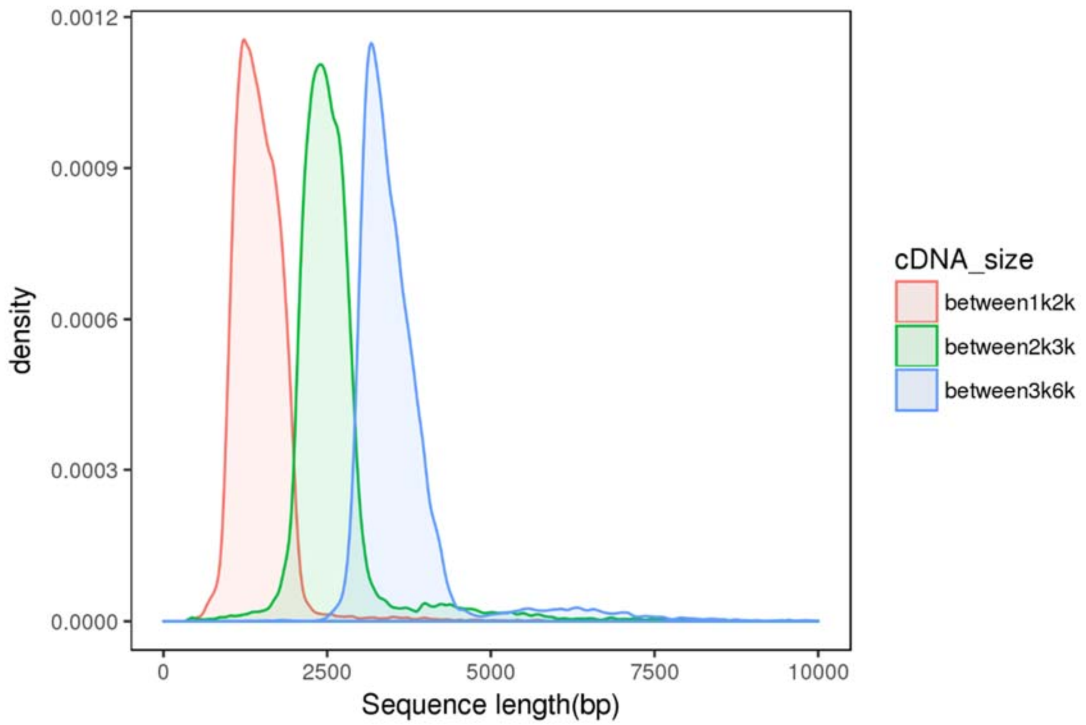

Figure S2. Consensus length density of *C. vestalis* unigenes obtained by PacBio Iso-Seq.

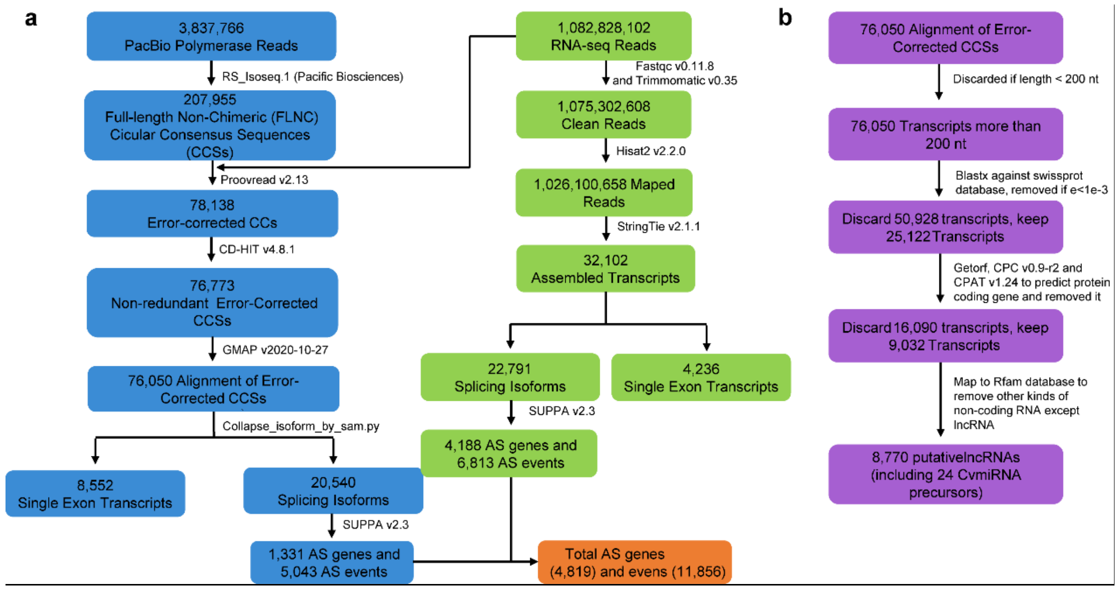

Figure S**3.** Computational pipeline for alterative splicing analysis and lncRNAs identification by combining Iso-Seq and RNA-Seq. (**a**) The workflow for the identification of alternative splicing by combining the data of Iso-Seq and RNA-Seq. The RNA-Seq data were mapped against the *C. vestalis* genome assembly CvesOGS1.0 by Hisat2 v2.2.0, and the transcripts were assembled using StringTie v2.1.1. In addition, the circular consensus sequences (CCSs) were calculated from PacBio subreads and corrected using the proofreading software Proovread v2.13 combined with RNA-Seq data. Then, the non-redundant error-corrected CCSs were obtained by using CD-HIT v4.81. Furthermore, the non-redundant and corrected full-length non-chimeric CCSs (FLNC CCSs) were mapped against CvesOGS1.0 using GMAP v2020-10-27 with default parameters. Next, the mapped reads were sorted and filtered, followed by being collapsed into non-redundant splicing isoforms by using collapse_isoforms_by_sam.py (https://github.com/Magdoll/cDNA_Cupcake). AS was identified by the software SUPPA v2.3 based on the GTF files produced after Iso-Seq and RNA-SeqS data were mapped to the genome of *C. vestalis*. (**b**) The workflow for the identification of lncRNAs in *C. vestalis*.

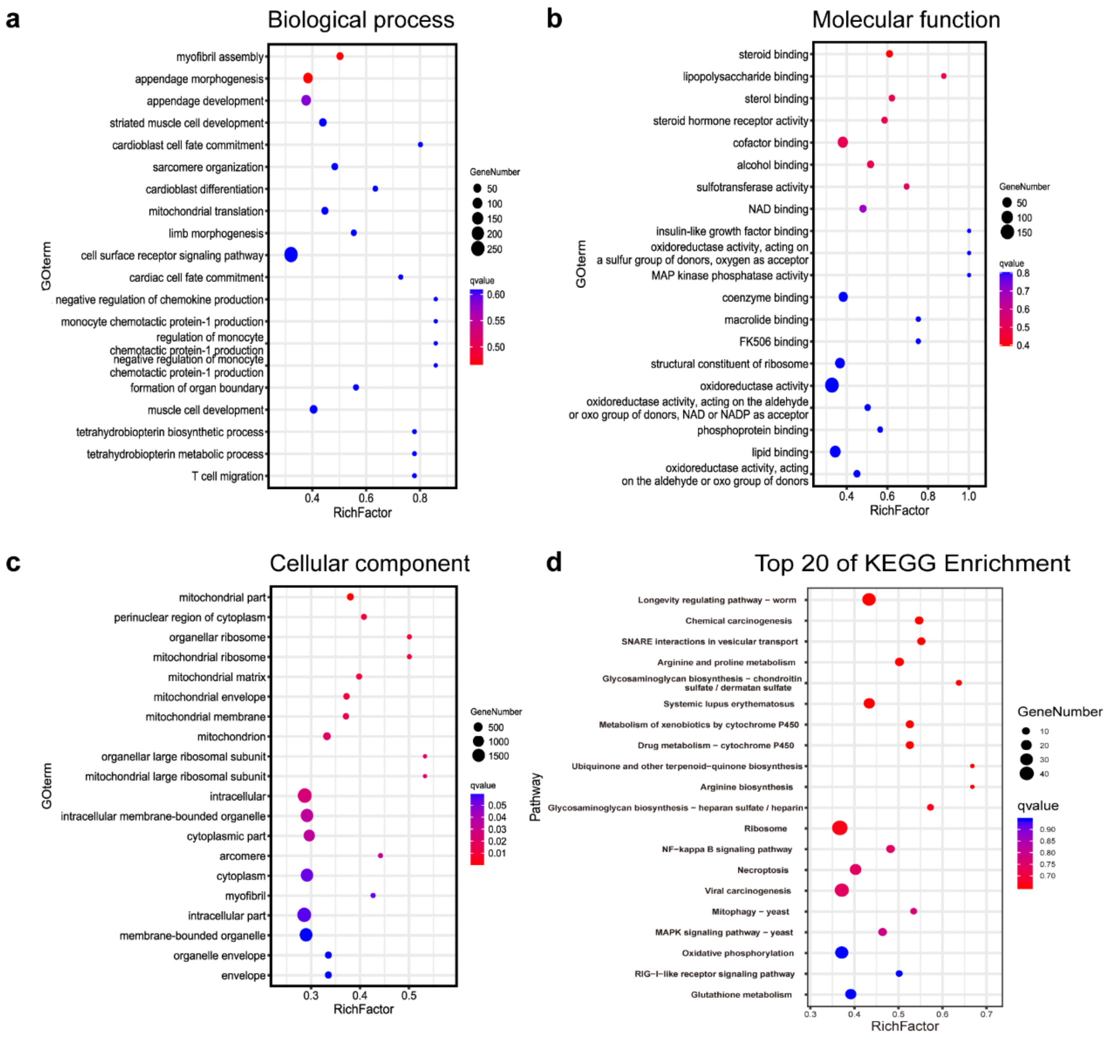

Figure S4. GO and KEGG annotation of putative targeted genes of lncRNA. The GO annotation include three subgroups: biological process (BP), cellular component (CC), and molecular function (MF). (**a**) The top 20 GO categories in biological process. (**b**) The top 20 GO categories in cellular component. (**c**) The top 20 GO categories in molecular function. (**d**) The top 20 KEGG enrichment pathways of putative target genes of lncRNA.

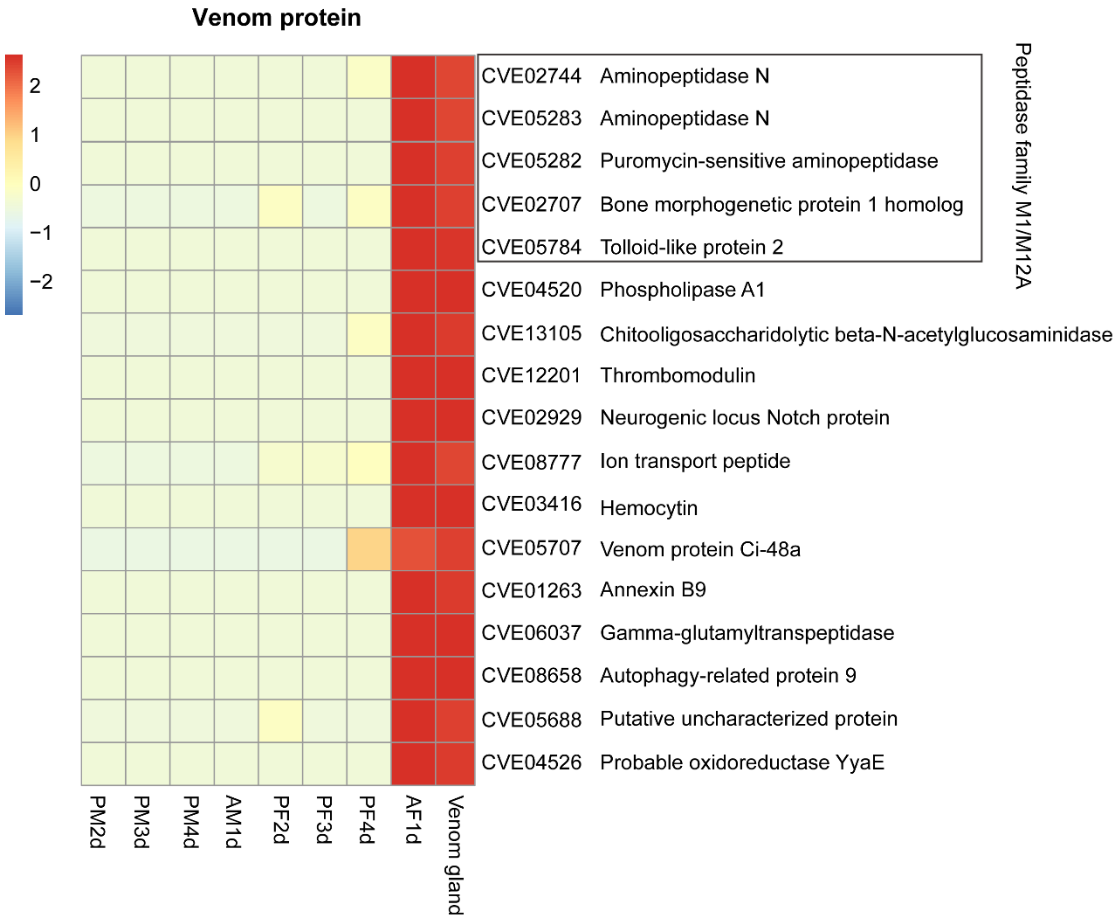

Figure S5. *C. vestalis* putative venom protein genes expression pattern.

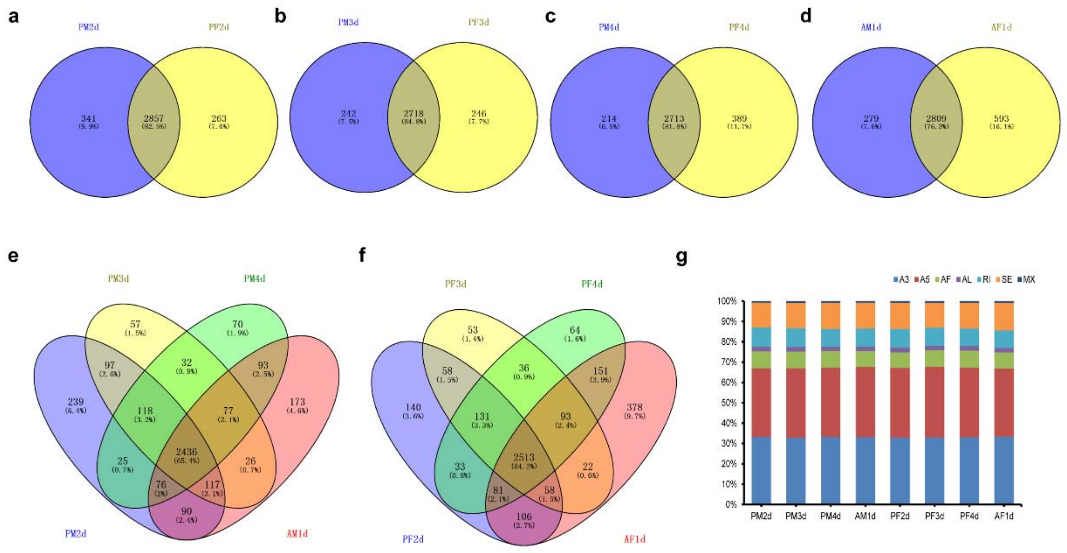

Figure S6. AS events identified in the *C. vestalis* genome. (**a**) Venn diagram of AS genes identified in PM2d and PF2d. (**b**) Venn diagram of AS genes identified in PM3d and PF3d. (**c**) Venn diagram of AS genes identified in PM4d and PF4d. (**d**) Venn diagram of AS genes identified in AM1d and AF1d. (**e**) Venn diagram of AS genes identified in different developmental stages of male in *C. vestalis*. (**f**) Venn diagram of AS genes identified in in different developmental stages of female in *C. vestalis*. (**g**) Distribution of the different AS events among developmental stages including those are common in all stages.

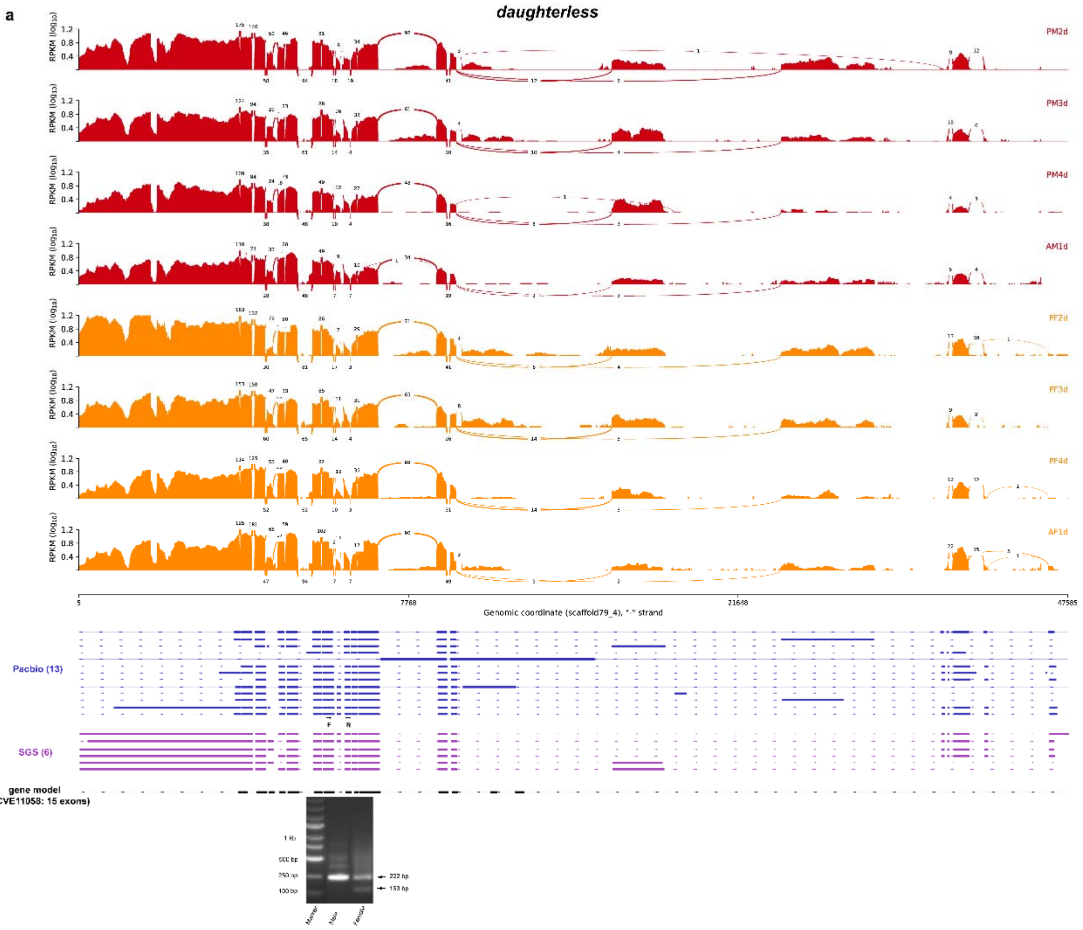
a
daughterless
RPKM (log10)
PM2d
PM3d
PM4d
AM1d
PF2d
PF3d
PF4d
AF1d
5
7768
21648
47585
Genomic coordinate (scaffold79_4), "-" strand
Pacbio (13)
F
R
SGS (6)
gene model
(CVE11058: 15 exons)
1 kb
500 bp
250 bp
100 bp
222 bp
153 bp
Marker
Male
Female

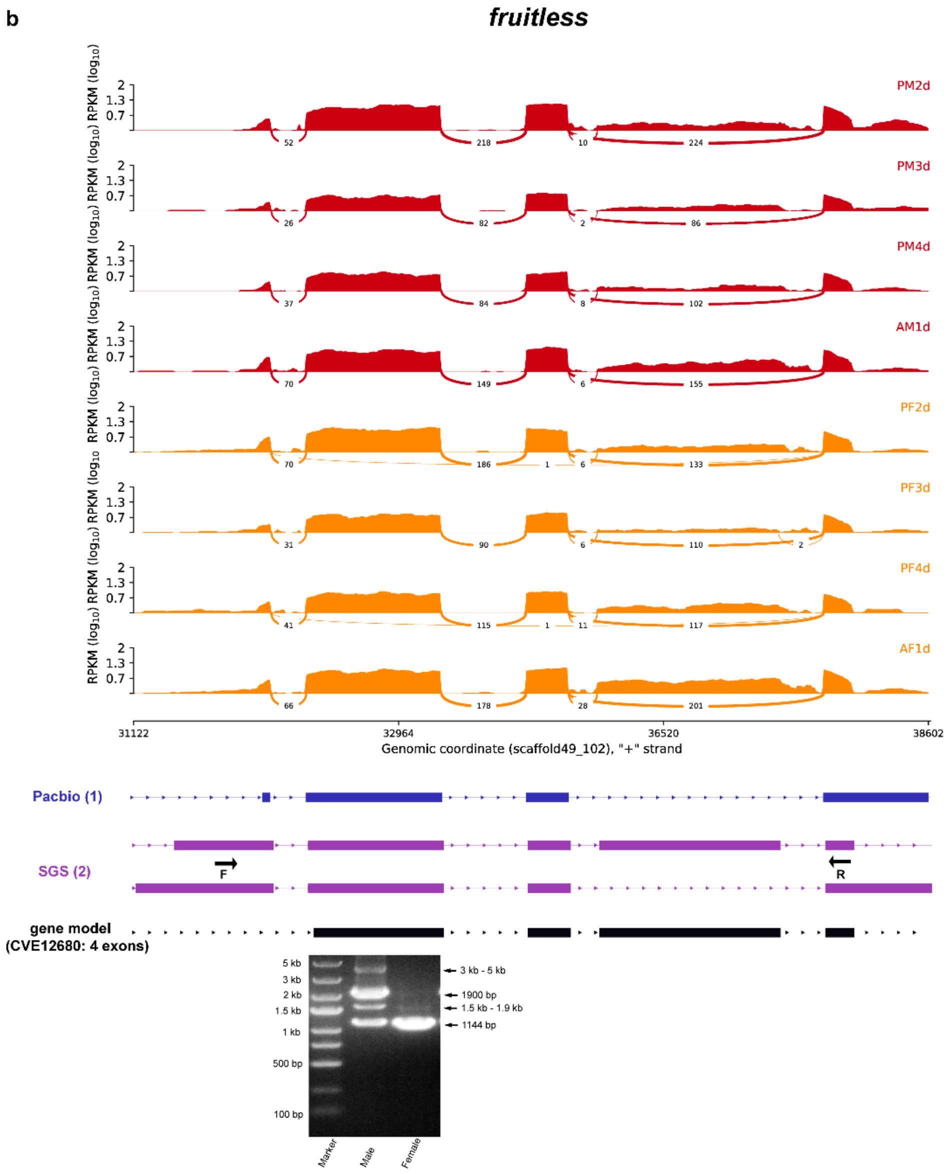
b
fruitless
PM2d
PM3d
PM4d
AM1d
PF2d
PF3d
PF4d
AF1d
RPKM (log10)
Genomic coordinate (scaffold49_102), "+" strand
Pacbio (1)
SGS (2)
F
R
gene model
(CVE12680: 4 exons)
5 kb
3 kb
2 kb
1.5 kb
1 kb
500 bp
100 bp
3 kb - 5 kb
1900 bp
1.5 kb - 1.9 kb
1144 bp
Marker
Male
Female

c *transformer-2*

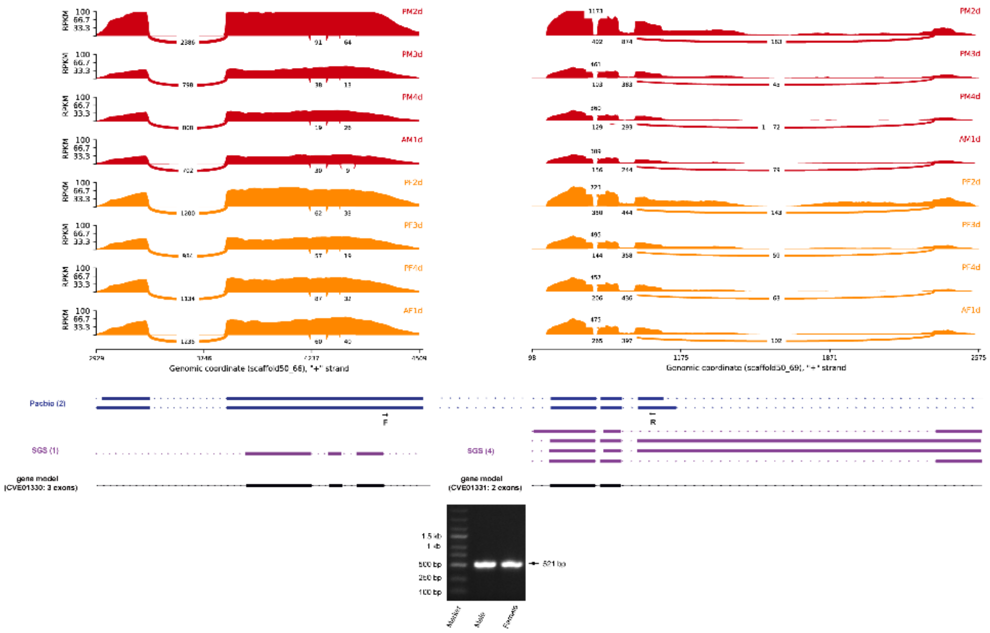

d *intersex*

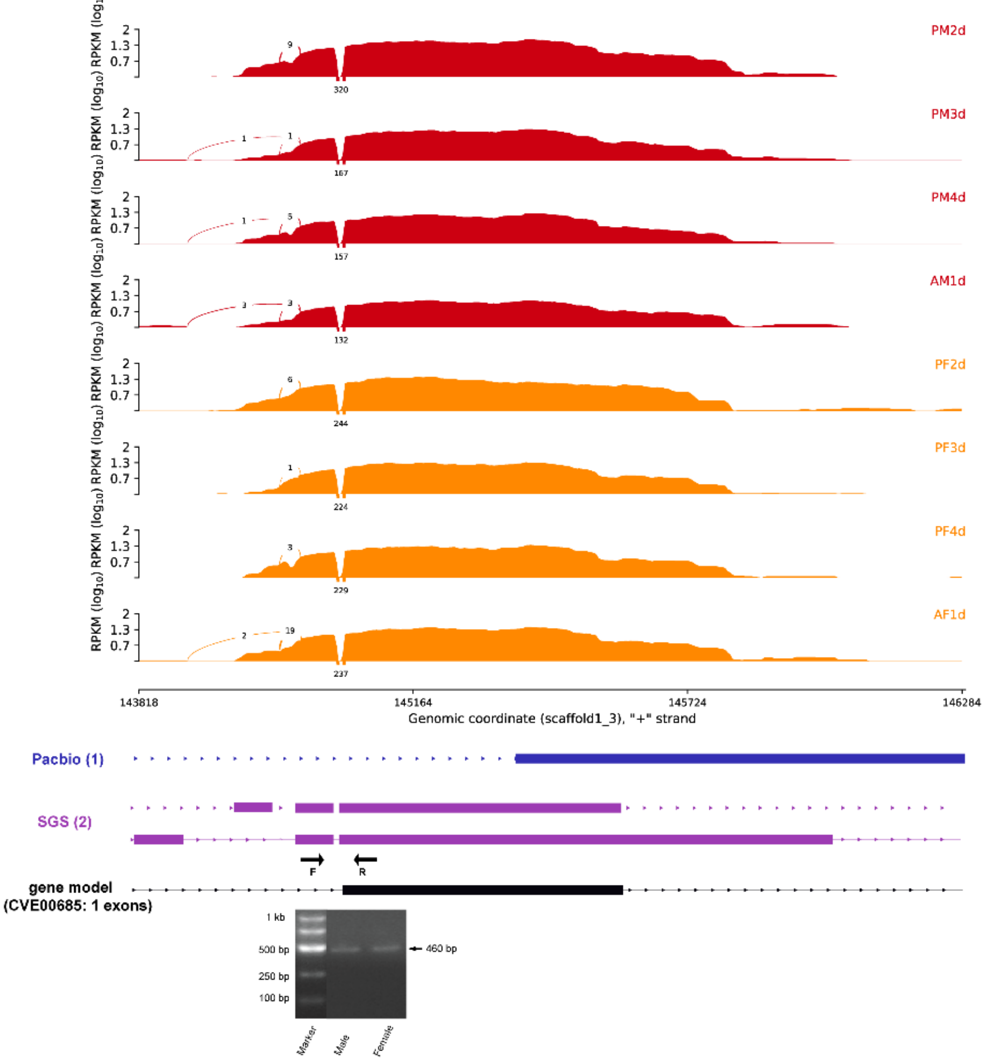

Figure S7. Alternative splicing events of four sex determining genes in *C. vestalis*. (**a**) Sashimi plot showing 19 alterative isoforms of *daughterless* gene in *C. vestalis*. (**b**) Sashimi plot showing 3 alterative isoforms of *fruitless* gene in *C. vestalis*. (**c**) Sashimi plot showing 7 alterative isoforms of *transformer-2* gene in *C. vestalis*. (**d**) Sashimi plot showing 3 alterative isoforms of *intersex* gene in *C. vestalis*. The thickness of curve in red and orange crossing two splicing junction sites (SJ) represents the coverage degree of Illumina RNA-Seq reads, and the number of junction reads is marked in the middle of the splicing site. The RNA coverage was given as the log-transformed reads per kilobase of transcript per million mapped reads (RPKM) value. Blocks represent exons and lines represent introns. The black rectangular box indicates sex-biased exons selected for RT-PCR verification. Forward and reverse primers used in RT-PCR were marked as F and R, respectively. SGS, second generation sequencing.
